# Simulating the mechanical stimulation of cells on a porous hydrogel scaffold using an FSI model to predict cell differentiation

**DOI:** 10.3389/fbioe.2023.1249867

**Published:** 2023-09-19

**Authors:** Pedram Azizi, Christoph Drobek, Silvia Budday, Hermann Seitz

**Affiliations:** ^1^ Chair of Microfluidics, Faculty of Mechanical Engineering and Marine Technology, University of Rostock, Rostock, Germany; ^2^ Department of Mechanical Engineering, Institute of Applied Mechanics, Friedrich-Alexander-University Erlangen-Nürnberg, Erlangen, Germany

**Keywords:** cell differentiation, tissue engineering, fluid-solid interaction, computational fluid dynamic, finite element analysis, *in silico*

## Abstract

3D-structured hydrogel scaffolds are frequently used in tissue engineering applications as they can provide a supportive and biocompatible environment for the growth and regeneration of new tissue. Hydrogel scaffolds seeded with human mesenchymal stem cells (MSCs) can be mechanically stimulated in bioreactors to promote the formation of cartilage or bone tissue. Although *in vitro* and *in vivo* experiments are necessary to understand the biological response of cells and tissues to mechanical stimulation, *in silico* methods are cost-effective and powerful approaches that can support these experimental investigations. In this study, we simulated the fluid-structure interaction (FSI) to predict cell differentiation on the entire surface of a 3D-structured hydrogel scaffold seeded with cells due to dynamic compressive load stimulation. The computational FSI model made it possible to simultaneously investigate the influence of both mechanical deformation and flow of the culture medium on the cells on the scaffold surface during stimulation. The transient one-way FSI model thus opens up significantly more possibilities for predicting cell differentiation in mechanically stimulated scaffolds than previous static microscale computational approaches used in mechanobiology. In a first parameter study, the impact of the amplitude of a sinusoidal compression ranging from 1% to 10% on the phenotype of cells seeded on a porous hydrogel scaffold was analyzed. The simulation results show that the number of cells differentiating into bone tissue gradually decreases with increasing compression amplitude, while differentiation into cartilage cells initially multiplied with increasing compression amplitude in the range of 2% up to 7% and then decreased. Fibrous cell differentiation was predicted from a compression of 5% and increased moderately up to a compression of 10%. At high compression amplitudes of 9% and 10%, negligible areas on the scaffold surface experienced high stimuli where no cell differentiation could occur. In summary, this study shows that simulation of the FSI system is a versatile approach in computational mechanobiology that can be used to study the effects of, for example, different scaffold designs and stimulation parameters on cell differentiation in mechanically stimulated 3D-structured scaffolds.

## 1 Introduction

Hydrogels are polymer networks that can hold large amounts of water, and their mechanical properties can be adjusted to match many native tissues (Blache et al., 2022). Their material characteristics have made them proper choices for producing scaffolds in tissue engineering. These hydrogel-based scaffolds form an artificial microenvironment that can mimic many properties of the native extracellular matrix (ECM) and respond to different stimuli similarly to the native ECM (Neves et al., 2020). Many cell activities in their microenvironment depend on mechanical stimulation. For example, organ formation, tissue regeneration, repair, and aging depend highly on the dynamic interaction between cells and their microenvironment (Iskratsch et al., 2014). The application of 3D hydrogel scaffolds in cartilage and bone tissue engineering for studying the repair processes both *in vitro* and *in vivo* is of great scientific interest (Song et al., 2020). The 3D structured scaffolds, which are modeled using computer-aided design (CAD), can be fabricated using additive manufacturing (AM) strategies. One printing technique within AM that has been utilized to create 3D structured scaffolds is the direct ink writing (DIW) method (Saadi et al., 2022). For example, DIW-printed scaffolds can be applied to repair and regenerate load-bearing bone defects (Fu et al., 2011; Deliormanli and Rahaman, 2012). The printed bio-scaffolds’ mechanical behavior is in good agreement with human bone and cartilage tissues, and they have outstanding biocompatibility (Saadi et al., 2022). Hydrogel scaffolds are designed to mimic biological structures, and since cells can be seeded into them homogeneously, they are great options for creating *in vitro* cell culture systems (Naveena et al., 2012). Bioreactors allow to create an environment for these cell cultures, which imitates the *in vivo* physiological conditions (Schulz and Bader, 2007). As an example of bioreactors’ application in tissue engineering, Meinert et al., 2017 created a novel bioreactor system to develop human cartilage neotissue promoted by mechanical stimulation in a controlled and monitored manner. Cell proliferation and differentiation processes inside the bioreactors are dependent on applied mechanical stimuli and, as a result, on the reconstructed cells’ microenvironment (Castro et al., 2020). The biological response of tissue and cells to mechanical stimulation is the subject of mechanobiology (Giorgi et al., 2016).

The use of *in silico* models in mechanobiology is a cost-effective method to broaden knowledge in tissue engineering and reduce the number of *in vitro* experiments. Moreover, computational mechanobiology is a powerful method to assess biological processes and provides valuable information on biophysical parameters that cannot be measured experimentally (Dolan et al., 2018). The finite element (FE) analysis and computational fluid dynamic (CFD) are the two main numerical methods applied in this field of study. The FE analysis has been performed to investigate the relationship between structural (porosity, pore size, pore architecture, etc.) and mechanical properties (stress, strain, elastic modulus, etc.) of regular and irregular scaffolds (Olivares et al., 2009; Gomez et al., 2016; Du et al., 2019; Arjunan et al., 2020). The CFD approach has been widely used to study scaffolds’ permeability and wall shear stress (WSS) caused by fluid flow inside the scaffolds (Ali and Sen, 2018; Ouyang et al., 2019; Zhianmanesh et al., 2019; Mahammod et al., 2020). In addition to CFD simulation of unseeded scaffolds, researchers have also utilized CFD to understand the influence of cell (or tissue) growth on the flow field surrounding the scaffold (Lesman et al., 2010; Guyot et al., 2015). However, mechanobiology is a multiscale and multiphysics problem. Hence, the fluid-structure interaction (FSI) simulation provides new opportunities for researchers to study mechanobiology (Giorgi et al., 2016).

Simulation of an FSI system can simultaneously analyze fluid and solid environments for tissue engineering applications. Tresoldi et al. (2017) conducted a two-dimensional axial-symmetric FSI simulation to evaluate mechanical stimulations acting on a scaffold system for vascular cells. They showed that FSI-computed working pressure and circumferential strains were in good agreement with the experimental values. In a recent work by Zhao et al. (2020) a multiscale FSI model was applied to evaluate the mechanical stimulation received by the cells in a perfusion bioreactor before (at day 0) and after tissue growth (at day 28). Their numerical investigation employed the FSI simulation at the microscale level (cell/ECM) at 12 locations in the scaffold, and the flow was modeled steady-state in the perfusion bioreactor. In the study of Ferroni et al. (2016) a 3D FSI micro-scale model of a porous scaffold was created to evaluate the WSS inside it. From a comparison of the computed WSS between the simple laminar flow model and FSI, they concluded that implementing an FSI model is mandatory to accurately predict the interaction between media and the scaffold during mechanical stimulation. The influence of structural parameters of regular scaffolds on mechanical stimulation and mass transport was studied by Malve et al. (2018) using the FSI simulation. Their investigation reported that cells should be seeded in the central regions of the scaffold to avoid high WSS values near the outer edges of the scaffold and to have uniform nutrient distribution within it. Fu et al. (2021) used a two-way method to simulate the FSI system and investigate the effect of the structural design of the scaffolds on the WSS on the surface of the deformable cells. In the computational study by Zhao et al. (2015) osteoblasts were modeled as cells attached to the scaffold or as cells bridged within the scaffold pores. Their FSI results found that fluid flow stimulated bridged cells more significantly than attached cells. In their computational analysis, similar to the study by Zhao et al. (2020) FSI simulation was employed in the microscale level for stimulation by perfusion under steady-state fluid flow.

Numerical methods are also utilized to predict the cell phenotype in tissue differentiation. Computational models of tissue differentiation consider mechanical loads that produce biophysical stimuli, including stress, strain, fluid flow, pressure, electrical potential, etc.(Prendergast et al., 2010,356). Prendergast et al. (1997) presented a mechano-regulationtheory in which mesenchymal stem cells’ fate is regulated by the combined biophysical stimuli(S) of tissue shear strain and fluid flow. Researchers used this theory to predict tissue phenotypes, especially with poroelastic FE analysis (Huiskes et al., 1997; Lacroix et al., 2002; Isaksson et al., 2006; Byrne et al., 2007; Milan et al., 2010; Koh et al., 2019; Perier-Metz et al., 2020). However, some other studies have adopted the mentioned mechano-regulation concept to alternative numerical methods (Olivares et al., 2009; Sandino and Lacroix, 2011; Hendrikson et al., 2017; Castro and Lacroix, 2018). Castro and Lacroix (2018) modeled the scaffold and its local environment as poro-hyperelastic materials. Their computational study evaluated the mechanical stimuli for different geometry models (CAD- and μCT-derived models) under unconfined and confined compression using nine FE models. Two separate FE simulations were performed in the investigation by Sandino and Lacroix (2011). In one FE analysis, the octahedral shear strain (OSS) was computed as a result of compressive strain, and in the other FE model, steady-state perfusion fluid flow inside the scaffold was simulated to obtain the WSS. From the computed values of WSS and OSS, the mechano-regulatory stimulus (S) was computed at each element, and consequently, the differentiated tissues were predicted within the scaffold. Olivares et al. (2009) calculated the initial stimuli sensed by the cells by analyzing two different solid and fluid phases. They computed OSS using a linear elastic FE analysis of the scaffold and WSS using a steady-state CFD simulation within the pore volume to obtain the mechanical stimuli. Hendrikson et al. (2017) also studied the influence of compressive loads on cell differentiation of different scaffold architectures by employing a combination of FE and CFD simulations. Their study concentrated on the influence of the additive manufactured scaffold architecture on the stress and strain distribution and, thus, on the calculated mechanical stimuli.

In this study we present a transient one-way FSI model that considers mechanical and fluid dynamic influences on cells cultivated on a porous 3D scaffold. To the authors’ best knowledge, this is the first FSI-based model that can predict the phenotype of cells on the entire surface of dynamically stimulated scaffolds. In contrast to previous multiscale approaches that considered only a limited number of locations in the scaffold in a steady-state simulation (Zhao et al., 2015; Zhao et al., 2020), we simulated the complete scaffold transiently at the macroscale level to study the spatially resolved stimulation effects over the entire scaffold surface. This allows the numerical prediction of cell differentiation on the surface of a porous hydrogel scaffold as a result of mechanical compression stimulation. In a first parameter study, we used this model to analyze the differentiation of the cells on the surface of a 3D-structured hydrogel scaffold surface as a function of the load amplitude.

## 2 Materials and methods

### 2.1 Geometry

**FIGURE 1:**
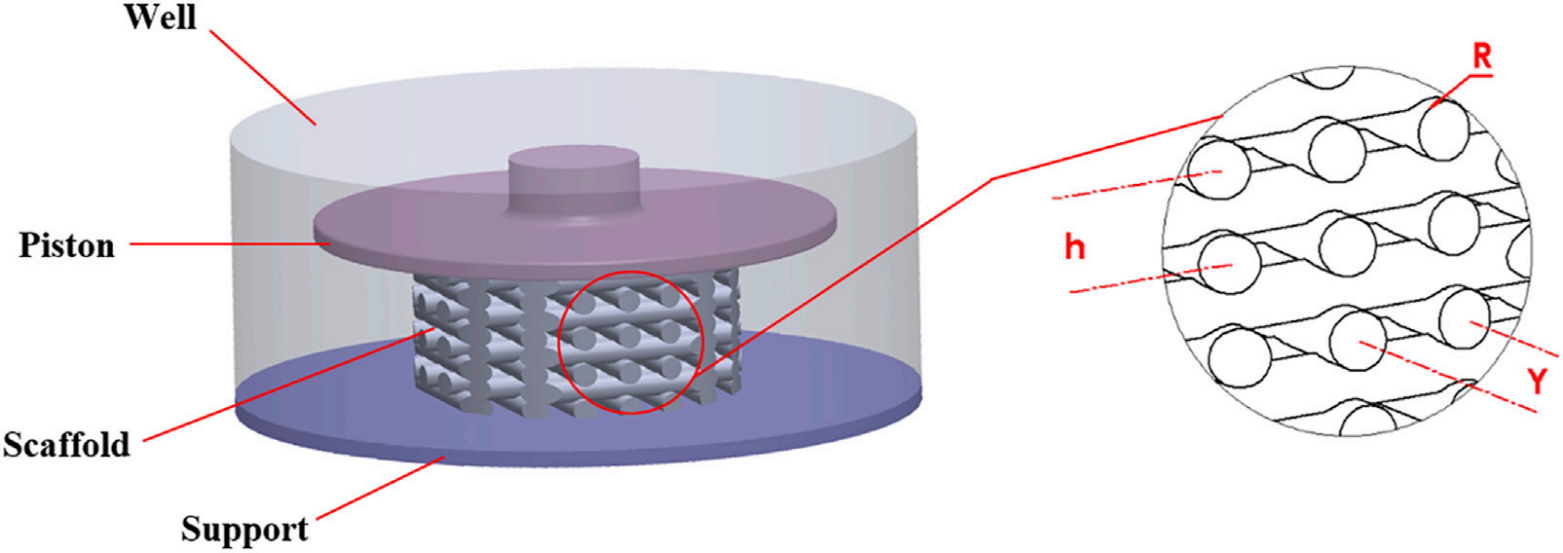
Geometry of the well, scaffold and piston (left side), scaffold details (right side).

A stimulation bioreactor was considered in which a small piston could move vertically to compress a scaffold inside a 12-well microwell plate. Accordingly, the diameter and height of the well were 21 and 8.5 mm, respectively, as shown in Figure 1. The top surface of the well is open so that the piston can move freely vertically to compress the scaffold.

The scaffold dimensions were chosen so that the scaffold could both be manufactured using DIW and fit into the 12-well microwell plate. The scaffold had a regular structure and was modeled with the CAD tool SOLIDWORKS (Dassault Systèmes SolidWorks Corporation, MA, United States). It had a height of 4.8 mm and a diameter of 10 mm. As shown in Figure 1, the geometrical parameters of the scaffold were defined as R = 0.35 mm (strand radius), Y = 1.4 mm (horizontal span), and h = 1.12 mm (vertical distance between two adjacent strands).

### 2.2 Mesh generation

**FIGURE 2:**
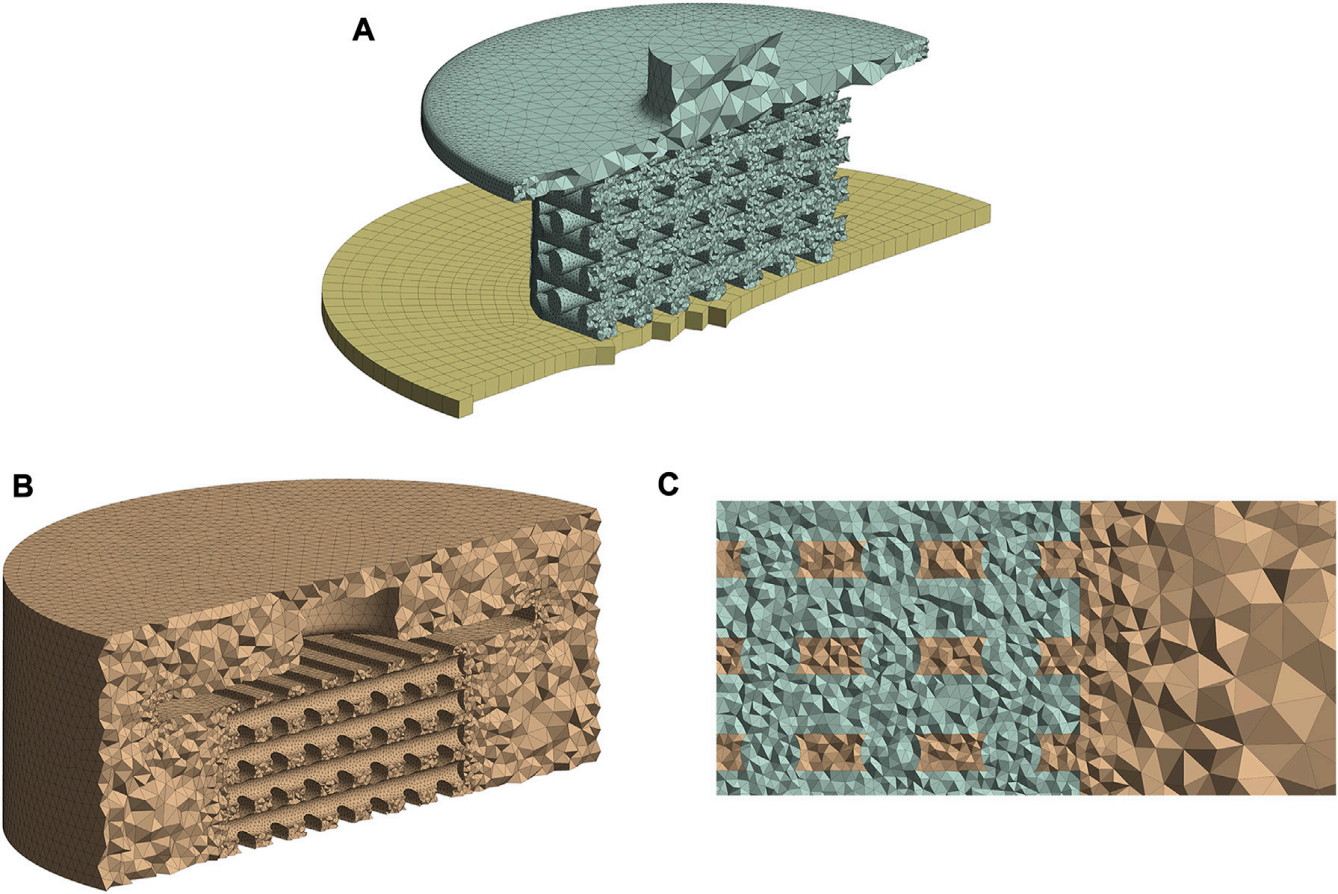
Mesh structures of the FE **(A)** and CFD **(B)** models. Conformal meshing between fluid and solid zones was applied to the scaffold surface **(C)**. All figure panels show section views of the numerical models.

To generate the mesh and run the simulations ANSYS 2020R2 (ANSYS Inc., PA, United States) was utilized. The entire structural and fluid domains were spatially discretized using ANSYS Meshing. During the mesh generation, particular attention was paid to the scaffold surface, as this region needed to be properly refined for the calculation of the mechano-regulatory forces. The mesh configurations of the FE and CFD models are shown in Figures 2A, B, respectively, where the areas with refined mesh can be recognized. Scaffold and piston were discretized using tetrahedral (Tet10) elements in the solid model while hexahedral elements (Hex20) were applied for the support (Figure 2A). The whole fluid domain was meshed using tetrahedral (Tet10) elements (Figure 2B). It can be seen from Figure 2C that the conformal meshing technique is applied between solid and fluid environments on the scaffold surface. As a result, FE and CFD nodes matched at each element on the scaffold surface, and calculating structural and fluidic properties at the same node was possible.

A mesh independence study was performed to understand the influence of element numbers on the simulation results (Supplementary Table S1 in Supplementary Material). Considering the mesh size influence on cell phenotypes prediction (Supplementary Table S2 in Supplementary Material), a mesh configuration of 7,80,094 FE model elements and 1,216,293 CFD elements (Medium mesh from Supplementary Table S1 in Supplementary Material) had good agreement with the finest studied mesh. Therefore, this mesh setup was chosen for all simulations in our study.

### 2.3 FE model

**FIGURE 3:**
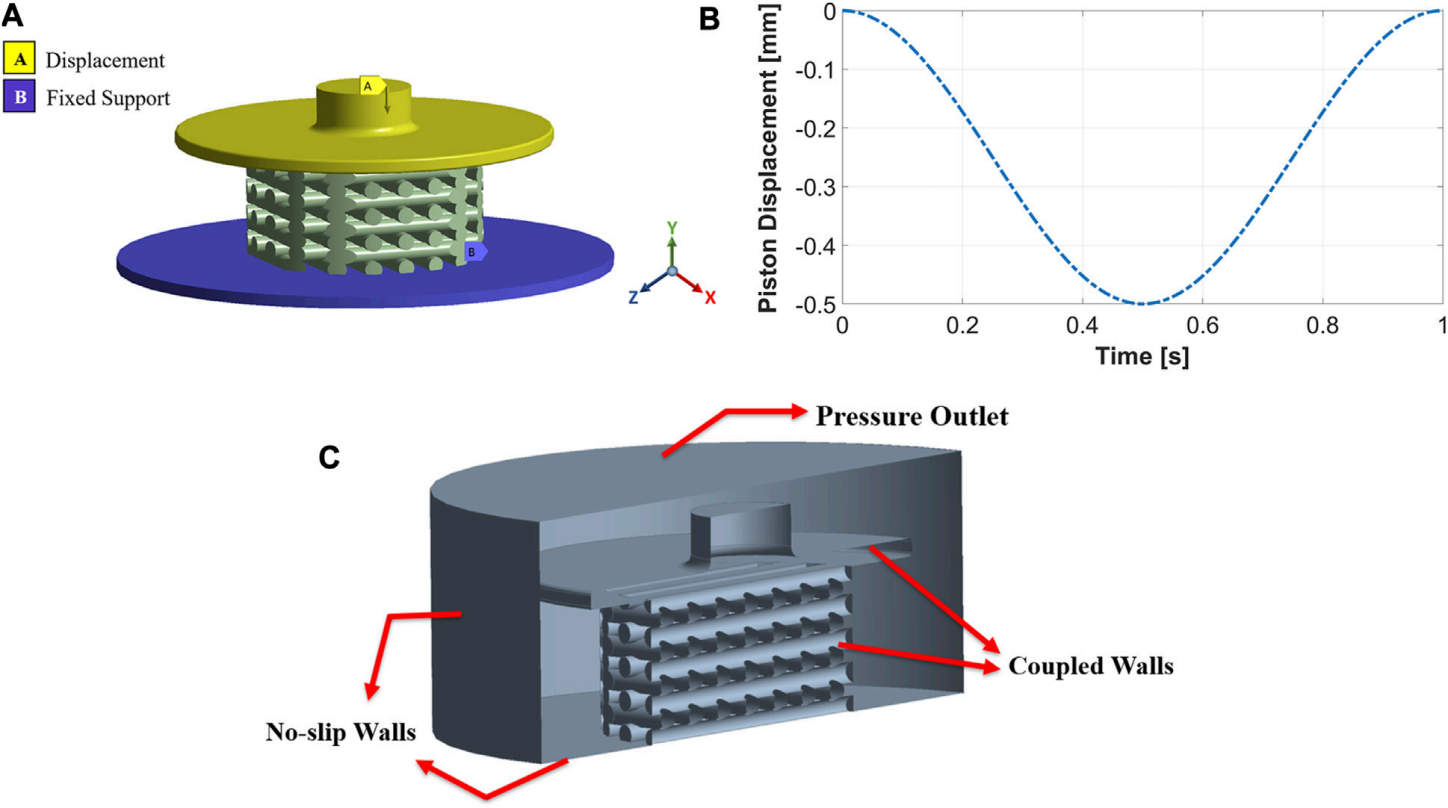
The FE model includes a movable piston on the top, support at the bottom, and a scaffold between the two **(A)**. Implemented compression load during one cycle for an amplitude of 10% **(B)**. Boundary conditions of the CFD model **(C)**.

The boundary conditions were determined in ANSYS Transient Structural to perform FE analysis. Figure 3A shows the three parts of the FE model: piston, scaffold, and support. The piston can move vertically (y direction) to compress the scaffold, while the support is fixed and cannot move or rotate in any direction. Both piston and support have frictional contact with the scaffold, and the vertical displacement of the piston was defined sinusoidally with a frequency of 1Hz, as shown in Figure 3B. Sinusoidal displacement with a frequency of 1 Hz was successfully applied in the study by Pioletti et al. (2003) to experimentally stimulate osteoblast-like cells. Moreover, previous studies also investigated mechanical stimulation in tissue-engineered cartilage, applying a dynamic compression load at 1 Hz (Mauck et al., 2000; Kisiday et al., 2004), because this frequency mimics the pace of the human gait (Salinas et al., 2018). We performed ten simulations with ten different compression amplitudes to study the influence of compression amplitude on cell differentiation. These values were 1%–10% of the original scaffold height with an interval of 1%. Previous *in vitro* studies of dynamic compressive loading on tissue-engineered cartilage confirms that amplitudes up to 10% improves biomechanical and biochemical properties (El-Ayoubi et al., 2011; Nebelung et al., 2012; Shahin and Doran, 2012; Salinas et al., 2018). Furthermore, Michalopoulos et al. (2012) found that seeded human mesenchymal stem cells (MSCs) differentiate toward osteogenesis on scaffolds stimulated with a cyclic compressive strain of 10%.

A pure oxidized alginate-gelatin (ADA-GEL) hydrogel was chosen as the scaffold material. Based on compression-tension experiments (Distler et al., 2021), we have previously shown that the hyperelastic response of pure ADA-GEL can be well captured by the one-term Ogden model (Ogden, 1972). We used the ANSYS hyperelastic material model in this study. To determine hyperelastic materials, a strain-energy density function (usually denoted as W ) must exist as a function of one of the strain or deformation tensors (Ansys^®^ Academic Research Mechanical (2020a)). The Lagrangian formulation of the strain-energy density function for determining strain or stress tensors can be found in Ansys^®^ Academic Research Mechanical (2020a). The general form of the Ogden model implemented in ANSYS is:

$$W=\sum_{i=1}^{N}\frac{\mu_{i}}{\alpha_{i}}\left({\bar{\lambda_{1}}}^{\alpha_{i}}+{\bar{\lambda_{2}}}^{\alpha_{i}}+{\bar{\lambda_{3}}}^{\alpha_{i}}-3\right)+\sum_{i=1}^{N}\frac{1}{d_{i}}{\left(J-1\right)}^{2i}$$ (1)

where *W* is the strain energy potential, $\lambda_{1},\lambda_{2}\;and\;\lambda_{3}$ are three principal stretch ratios which represent a measure of the deformation, *J* is the volume ratio which defines the ratio of deformed to undeformed volume of the material, $\mu_{i},\alpha_{i}\;and\;d_{i}$ are specified as material constants by users.

The set of material parameters was identified based on compression-tension experiments by Distler et al. (2021). We defined them for *N* = 1 as μ_1_ = −5.8 kPa and α_1_ = −1.3 on the assumption of a fully incompressible material behavior (d_1_ = 0). For fully incompressible materials, mixed u-P formulation had to be applied by ANSYS to get the solutions (Ansys^®^ Academic Research Mechanical, 2020b).

The material properties did not change during the loading cycle because the viscoelastic behavior of the scaffold was not considered. Therefore, only one loading cycle was simulated.

### 2.4 CFD model

Fluid flow inside the mechanically stimulated 3D-structured scaffold was modeled using ANSYS Fluent, which is based on the finite volume method (FVM). The fluid zones which received displacements from the structural model, i.e., piston and scaffold walls, were treated with a dynamic mesh approach. This approach combined smoothing based on the diffusion method with the remeshing method for all deforming zones. These parts of the fluid domain were coupled to the FE model and meshed as fluid-solid interface zones. No-slip wall boundary conditions were applied to the well’s bottom and cylindrical walls, as seen in Figure 3C. The fluid domain’s top surface was considered a pressure-outlet boundary with atmospheric pressure (Figure 3C).

The culture medium was modeled as a Newtonian, incompressible fluid with a density of 1,000 kg/ $m^{3}$ and a dynamic viscosity of $1.45\times 10^{-3}$ Pa s (Olivares et al., 2009). The flow regime was assumed to be laminar because the Reynolds number (Re), which was estimated based on pore dimension, was very small (Re < 3) even at maximum compression of 10%. The absolute criteria of scaled residual for continuity and velocity were defined < $10^{-4}$ to ensure convergent results.

### 2.5 FSI system

A 3D fluid-structure interaction (FSI) transient one-way model was used to analyze fluidic and mechanical properties of the regular scaffold. The FSI system was modeled based on a co-simulation strategy, in which a converged solution from the FE model was obtained at each coupling iteration. These results defined the CFD model’s new boundary conditions, i.e., deformation from the FE model induced fluid flow in the CFD model. The two physics solvers were coupled using ANSYS System Coupling. The co-simulation process was repeated at each simulation time step until the cycle of the compressive stimulation reached the end (i.e., after 1 s simulation time). Due to the physics of this FSI problem, a one-way FSI approach was sufficient instead of a two-way approach because the solid motion induced the fluid flow. Nevertheless, the fluid flow did not significantly affect the solid deformation. All simulations were performed on a Windows workstation with a 24-core processor and 256 GB RAM. Subsequently, the results of the FSI simulations were read with a MATLAB R2021a (Math Works Inc., MA, United States) script, and cell phenotypes were predicted using a mechano-regulatory algorithm described in chapter 2.6.

The accuracy of the results in the transient FSI model depends not just on the mesh size but also on choosing the appropriate time step size. In our study, the CFD model’s dynamic mesh method decides the time step size. According to ANSYS Fluent User’s Guide, Release 2020 R2, the relative mesh motion should not exceed the smallest element. This condition can be written as:

$$∆t<\;\frac{∆s}{V_{\mathrm{Max}}}$$ (2)

where ∆t is the maximum chosen time step size, ∆s is the minimum element length, and $V_{\mathrm{Max}}$ is the maximum expected velocity of the moving mesh.

Piston speed, mesh size, and compression amplitude are the three parameters influencing choosing time step size. The piston movement influences $V_{\mathrm{Max}}$ in Eq. 2, where faster piston movement (or higher piston frequency) leads to smaller possible time step sizes. Furthermore, mesh size and compression amplitude affect the term ∆s in Eq. 2. For example, if a small element size or high compression amplitude were chosen, the time step size must be smaller.

Taking the criteria of Eq. 2 into account, three different time step sizes were considered to assess their influence on the simulation results for a compression of 10%: large (0.004 s), medium (0.002 s), and small (0.001 s) time step sizes. Comparing the average and maximum values of OSS and WSS indicated that the three chosen time intervals result in almost similar structural and fluidic behavior. Moreover, the relative errors in cell phenotype prediction (bone, cartilage, and fibrous cells) of the large time step size compared with the small time step size were calculated at three sample simulation times. The relative error was less than 2% in all examined simulation times. As a result, the large time step size (0.004 s) was applied to perform the simulations with a lower computational cost.

### 2.6 Evaluation of mechanical stimuli

A German orthopedic surgeon, Friedrich Pauwels, related the biophysical stimuli to MSCs’ fate (Pauwels, 1960; Prendergast et al., 2010, 360–361). He hypothesized that deformation leads to the differentiation of MSCs into fibrous tissue, while hydrostatic compression gives rise to the differentiation of MSCs into cartilage; a mixture of these stimuli leads to fibrocartilage tissue (Prendergast, 2004, 119–120; Prendergast et al., 2010, 360–361). Based on the ideas of Pauwels, Prendergast et al. (1997) developed a mechano-regulation theory that proposed that the fate of MSCs is controlled by strain and fluid flow. A modified version of this theory (Olivares et al., 2009; Sandino and Lacroix, 2011; Hendrikson et al., 2017) which substitutes fluid velocity with wall shear stress (WSS), has been implemented in the current study to compute the mechano-regulatory stimulus as:

$$S=\frac{OSS}{a}+\frac{WSS}{b}$$ (3)

where *S* is the stimulus, *OSS* is the octahedral shear strain, *WSS* is wall shear stress, and constants *a* and *b* are equal to 0.0375 and 10 mPa (Olivares et al., 2009; Sandino and Lacroix, 2011; Hendrikson et al., 2017). The tissue phenotype was predicted by computing the stimulus (S) and its classification according to the modified mechano-regulation theory (Olivares et al., 2009; Sandino and Lacroix, 2011; Hendrikson et al., 2017): If S≤0.01 , the stimuli were too low, and no tissue differentiation was predicted; If 0.01<S≤1 , bone tissue differentiation was predicted. If 1<S≤3 , cartilage tissue differentiation was predicted. If 3<S≤6 , then fibrous tissue differentiation was predicted and if S>6 , the stimuli were too high, and no tissue differentiation was predicted.

The OSS was computed from the FE model as follows:

$$OSS=\frac{2}{3}\;\sqrt{{\left(\varepsilon_{1}-\varepsilon_{2}\right)}^{2}+{\left(\varepsilon_{2}-\varepsilon_{3}\right)}^{2}+{\left(\varepsilon_{3}-\varepsilon_{1}\right)}^{2}}$$ (4)

where $\varepsilon_{1}$ , $\varepsilon_{2}$ and $\varepsilon_{3}$ are the elastic principal strains and they are defined as:

$$\varepsilon_{i}=\lambda_{i}-1\;\left(i=1:3\right)$$ (5)

where $\varepsilon_{i}$ is the principal strain in the *i*th direction and $\lambda_{i}$ is the principal stretch ratio in the *i*th direction.

As mentioned in Section 2.3, the material model of the scaffold is not time-dependent because the viscoelastic properties of the scaffold are not considered. Therefore, the computed OSS values do not differ during successive loading cycles.

The WSS on the scaffold surfaces were calculated from the laminar CFD model as follows:

$$\text{WSS}=\mu \frac{\partial u}{\partial n}$$ (6)

where μ is the dynamic viscosity and $\frac{\partial u}{\partial n}$ is the fluid normal velocity gradient at the wall.

The two output parameters, OSS and WSS, had to be computed at the same element nodes to calculate S. Therefore, as mentioned in Section 2.2, a conformal meshing technique was applied to match the element nodes of the FE and CFD models. The FE and CFD results were matched at the same nodes using a MATLAB script; thus, the stimuli could be calculated. Since we developed a transient FSI model with dynamic loading conditions, different configurations of cell phenotypes on the scaffold surface could be calculated at each position of the moving piston (i.e., at each simulation time point). To predict tissue phenotypes after a single complete loading cycle, the following calculations were performed using MATLAB: First, the average value of stimuli ( $S_{avg}$ ) at each simulation time step was calculated based on area-weighted average values of wall shear stress over the entire scaffold surface ( ${WSS}_{avg}$ ) and the arithmetic mean of the octahedral shear strain of all the FE nodes on the scaffold surface ( ${OSS}_{avg}$ ). Then, the maximum value of $S_{avg}$ occurring during the loading cycle and its corresponding time step were determined. The tissue phenotypes were analyzed at this time step. This evaluation was repeated for all other loading conditions.

## 3 Results

**FIGURE 4:**
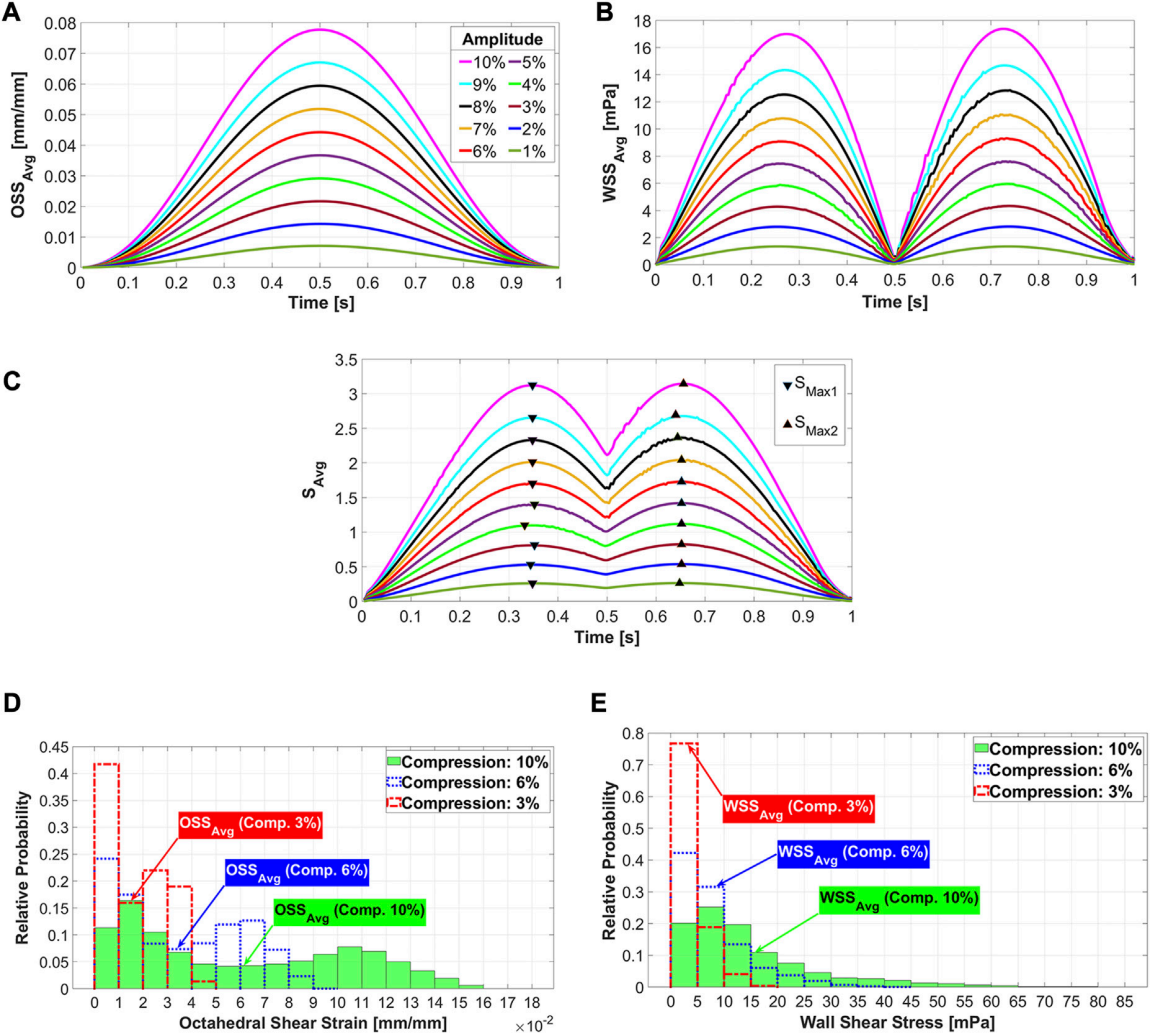
Average octahedral shear strain **(A)** and area-weighted averaged wall shear stress **(B)** during the loading cycle for different compression amplitudes. The average values of stimuli ( $S_{avg}$ ) as a function of averaged values of OSS and WSS **(C)**. The triangular signs show maximum values of $S_{avg}$ when the piston moves downwards ( $S_{\mathrm{Max}1}$ ) and when it moves upwards ( $S_{\mathrm{Max}2}$ ). Distribution of octahedral shear strain **(D)** and fluid wall shear stress **(E)** on the scaffold surface for three exemplary compression amplitudes of 3%, 6%, and 10% are reported using histograms.

In the FSI simulation of a dynamic mechanical stimulation process, the prediction of the cell phenotype on the scaffold surface changes at each simulation time point (piston position), as explained in Section 2.6. Figures 4A–C show the results used to determine the simulation time points at which the cell phenotypes were predicted. Figure 4A shows that ${OSS}_{avg}$ depend on the compression amplitudes. It can be seen from this figure that as the compression amplitude increased, the ${OSS}_{avg}$ also grew, and simultaneously it shows that the maximum values of ${OSS}_{avg}$ occurred when the piston was in its lowest position ( t=0.5 s ). As shown in Figure 4B, ${WSS}_{avg}$ gradually rose with increasing compression amplitude. The ${WSS}_{avg}$ ranged from 0 to 1.35 mPa for 1% compression and from 0 to 17.4 mPa for 10% compression. Figure 4C shows the calculated stimuli for different compression amplitudes based on ${WSS}_{avg}$ and ${OSS}_{avg}$ during the entire loading cycle. The $S_{avg}$ curves in Figure 4C are the outputs of Eq. 3, where ${WSS}_{avg}$ (Figure 4B) and ${OSS}_{avg}$ (Figure 4A) are the inputs of this equation. The two maxima of $S_{avg}$ were considered to predict the cell phenotypes at their respective simulation times: during the piston’s downward ( $S_{\mathrm{Max}1}$ ) and upward movements ( $S_{\mathrm{Max}2}$ ). The prediction of cell differentiation at the two time points mentioned did not show any significant difference. However, $S_{\mathrm{Max}2}$ values were slightly higher; therefore, these simulation time points were chosen to predict the differentiation of the cells at different compression amplitudes in our study. Figures 4D, E report the distribution of OSS and WSS on the scaffold surface for the compressions of 3%, 6% and 10% at the simulation time points when $S_{\mathrm{Max}2}$ occurs. These figures also highlight the OSS and WSS intervals, where ${OSS}_{avg}$ and ${WSS}_{avg}$ occur at the respective sample compressions. From these histograms, it can be seen that OSS and WSS were distributed in a wider range with increasing compression amplitude. This means that both parameters influencing mechanical stimulation increase with increasing compression amplitude.

**FIGURE 5:**
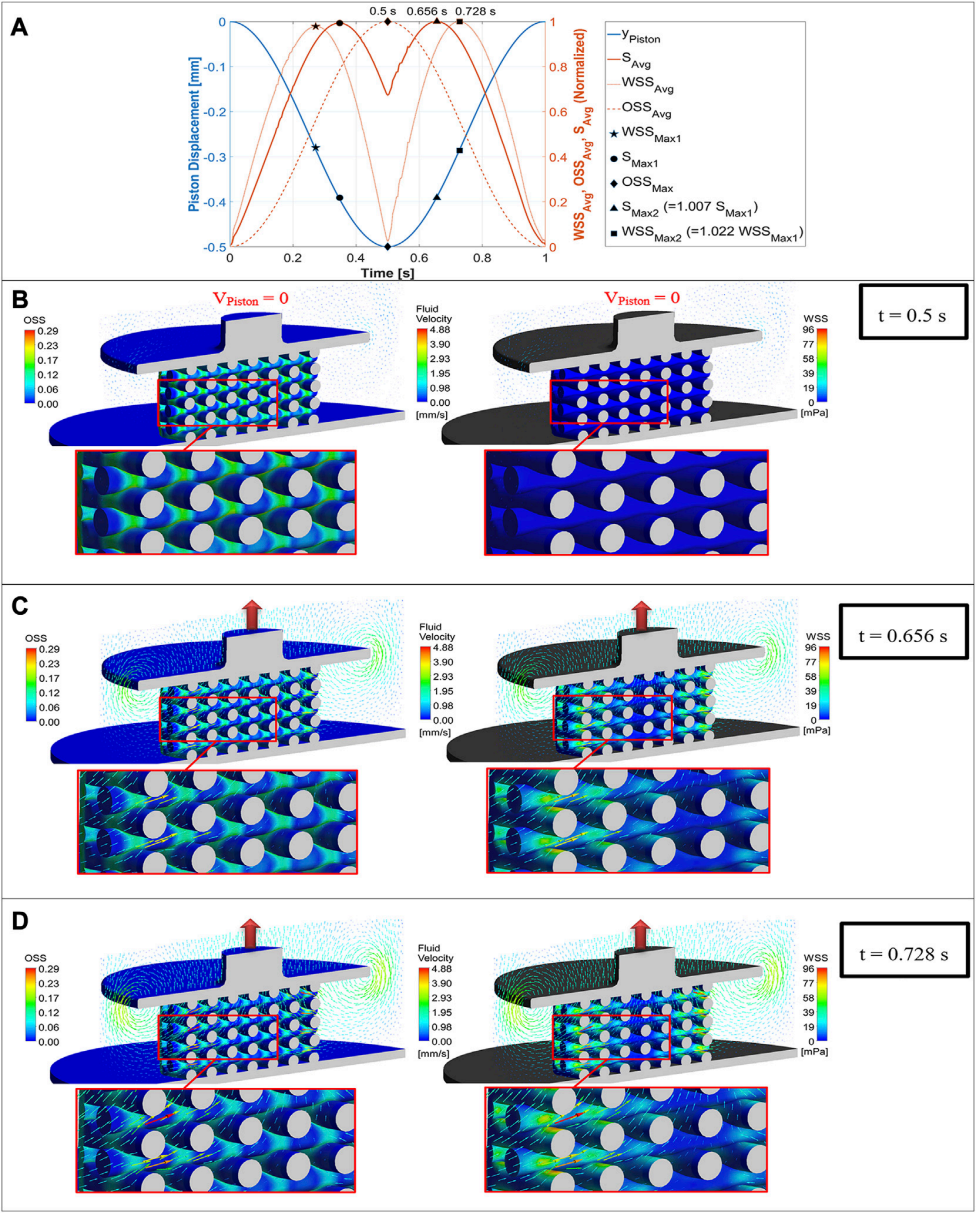
Mechano-regulatory stimuli (solid red curve) based on average values of WSS (dotted curve) and OSS (dashed curve) plotted versus simulation time along with the displacement curve of the piston (blue curve) for one loading cycle **(A)**, OSS distribution on the scaffold surface (left) compared with WSS distribution (right) for a compression amplitude of 10% at three different simulation time points (piston positions), when the maximum value of OSS **(B)**, the maximum value of $S_{avg}$ **(C)**, and the maximum value of WSS **(D)** occurred. OSS, WSS, and fluid velocity’s color scales remain unchanged in all cases.

Figure 5 shows both the fluid flow field and mechanical deformation of the scaffold during stimulation with a compression amplitude of 10% at three different simulation time points. The piston displacement curve as well as the three normalized curves of ${WSS}_{avg}$ , ${OSS}_{avg}$ and $S_{avg}$ during a loading cycle are shown in Figure 5A. The maxima of ${WSS}_{avg}$ , ${OSS}_{avg}$ , and $S_{avg}$ are marked not only on the corresponding curves but also on the piston displacement curve. This allows the piston position to be detected at these maxima. In Figures 5B–D, uniform color scales were used for the computed physical quantity OSS, WSS and fluid velocity respectively to facilitate comparison of these figures. The left sides of Figures 5B–D show the OSS distribution on the scaffold surface induced by the mechanical deformation at the indicated time points, while the right sides depict the flow-induced WSS distribution on the scaffold surface. The velocity vectors on the left and right sides of Figures 5B–D are the same and have an identical color scale. Figure 5B illustrates the fluid flow and mechanical behavior of the scaffold at t=0.5 s (diamond sign in Figure 5A), where the piston reached its lowest position and ${OSS}_{avg}$ rose to its maximum value. It can be seen from Figure 5B that fluid velocity has been reduced significantly, which is why the velocity vectors are too small to be visible. The left side of Figure 5B shows the OSS distribution on the scaffold surface, where it reached the largest values at the intersections of the strands. On the other hand, the WSS values at the surface of the strands were almost near zero due to the low fluid velocity gradients near the scaffold surface (right side of Figure 5B). Figure 5C displays fluid flow and mechanical behavior of the scaffold at t=0.656 s (triangle sign in Figure 5A), where $S_{avg}$ peaked during upward movement of the piston. The direction of the velocity vectors inside the scaffold pores and the direction of the vortex near the outer edges of the piston indicate the upward movement of the piston (Figure 5C). The left side of Figure 5C shows that the OSS on the scaffold surface was again highest at the intersections of the strands, but the values were lower than the OSS values in the same areas of the scaffold in Figure 5B. As can be seen on the right side of Figure 5C, the WSS was higher at areas with higher velocity. Figure 5D depicts the fluid flow and mechanical behavior of the scaffold at t=0.728 s (square sign in Figure 5A), when the piston moved upward and ${WSS}_{avg}$ reached its maximum. It is apparent from Figure 5D that the OSS values at the intersections of the strands were smaller than OSS values at the same surfaces in Figures 5B, C. The right side of Figure 5D reports that magnitude of the velocity vectors and the WSS reached higher values than for the previous piston positions, as was also expected based on the ${WSS}_{avg}$ curve in Figure 5A.

**FIGURE 6:**
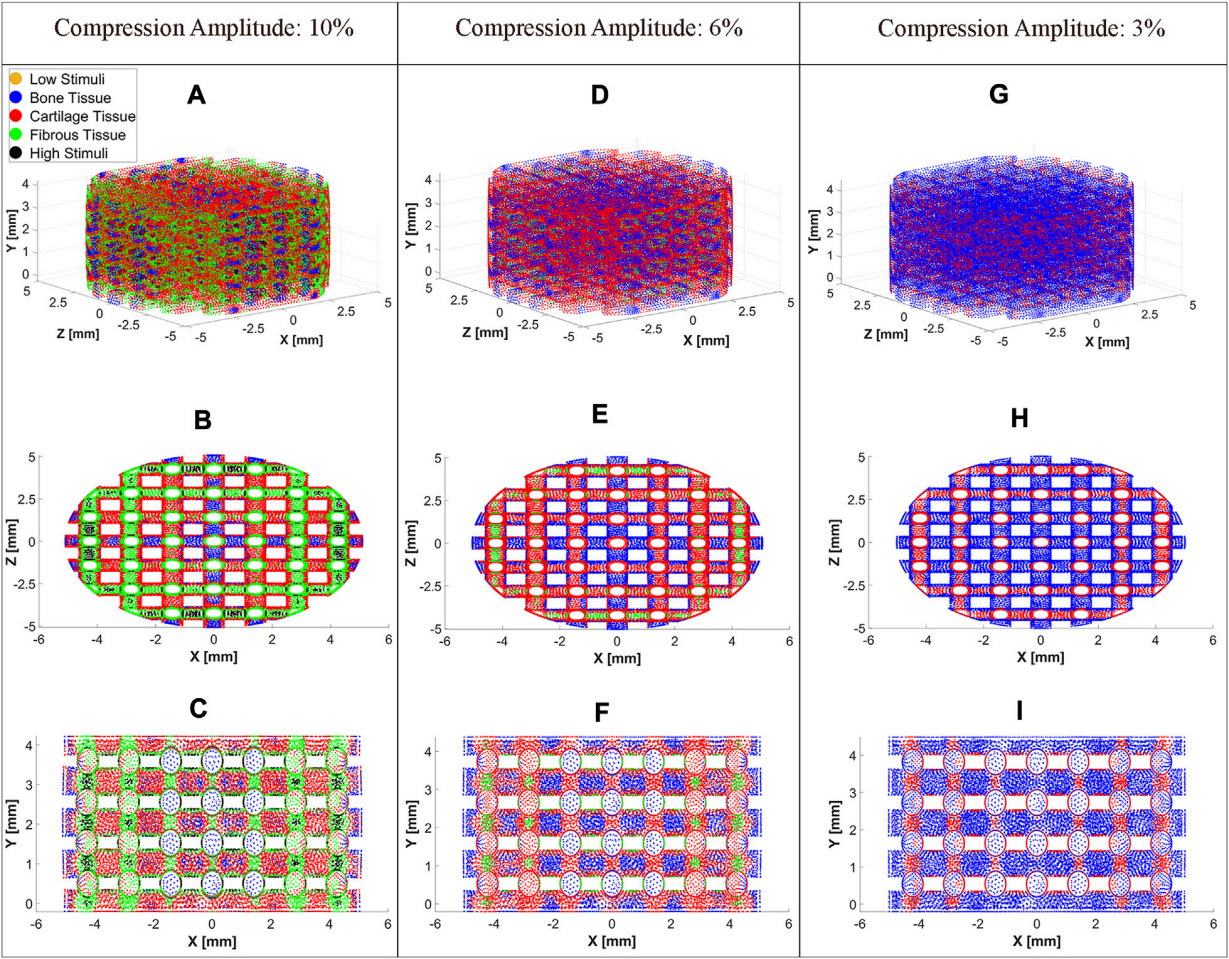
Cell differentiation on the scaffold surface at a compression amplitude of 10% **(A)**, 6% **(D)**, and 3% **(G)**. Top view (XZ Plane) of cell differentiation on the scaffold surface at a compression amplitude of 10% **(B)**, 6% **(E)**, and 3% **(H)**. Side view (XY Plane) of cell differentiation on the scaffold surface at a compression amplitude of 10% **(C)**, 6% **(F)**, and 3% **(I)**. The cell differentiation was predicted at t=0.656 s , when $S_{\mathrm{Max}2}$ occurred.

The cell distributions on the scaffold surface for the three exemplary compression amplitudes 3%, 6%, and 10% are shown in Figure 6 at t=0.656 s , when $S_{avg}$ peaked during upward movement of the piston ( $\left.S_{\mathrm{Max}2}\right)$ . For each compression amplitude, the 3D view of the scaffold is followed by the corresponding top and side views. Each mesh node represents a cell phenotype predicted using the mechano-regulatory theory. A compression amplitude of 10% resulted in bone, cartilage, and fibrous phenotypes. While these phenotypes dominated, the stimuli in a small scaffold surface area were too high to induce cell differentiation, as depicted in Figure 6A and the following top view (Figure 6B) and side view (Figure 6C). At a dynamic compression of 6%, cell differentiation towards cartilage and bone was predominantly induced, while on a small area of the scaffold surface, differentiation towards fibrous cells was stimulated (Figures 6D–F). Finally, a compression amplitude of 3% led to differentiation into bone and cartilage cells, with a greater proportion of bone cells compared to cartilage cells, as depicted in Figures 6G–I.

**FIGURE 7:**
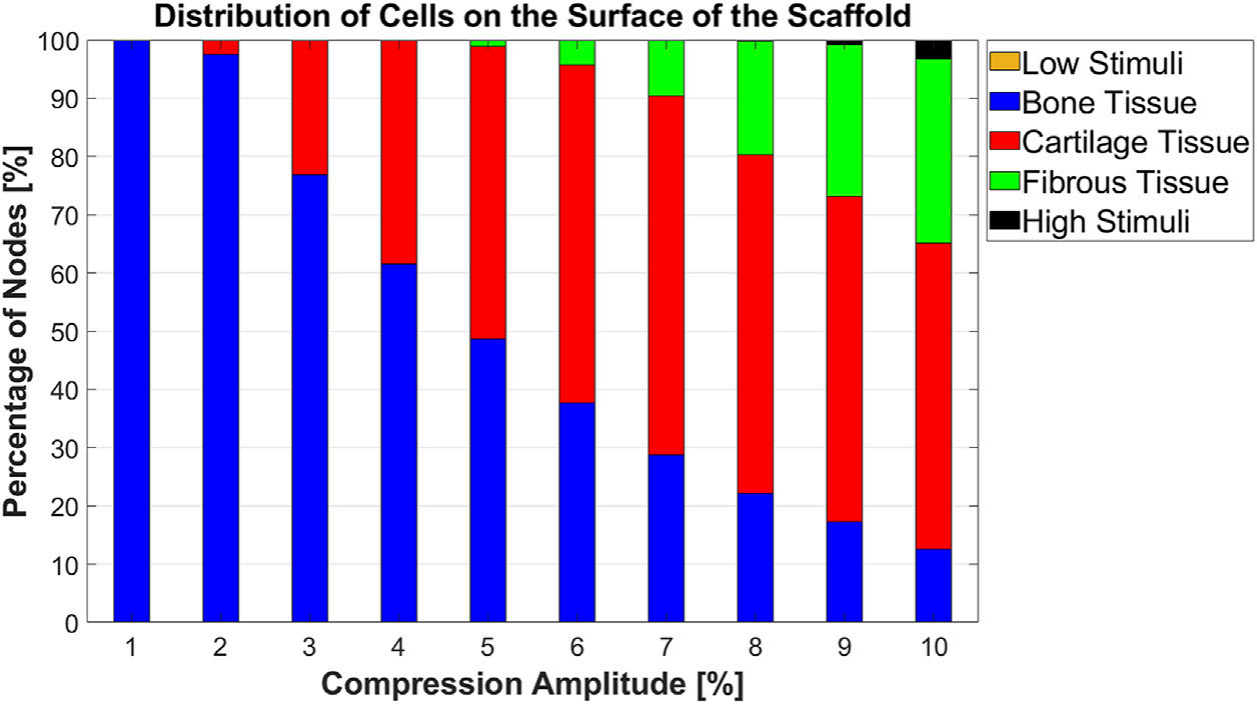
Percentage distribution of cell phenotypes on the scaffold surface for 10 different compression amplitudes.

An overview of the cell differentiation for the compression range between [1% 10%] with an interval of 1% is depicted in Figure 7. It can be seen from this graph that with increasing compression amplitude, bone cell differentiation decreased. Cartilage cell differentiation rose from 2% to 7% compression and decreased slightly thereafter. From 5% to 10% compression, a fibrous cell phenotype was predicted to develop on larger areas of the scaffold with increasing compression amplitude. At high compressions of 9%–10%, the stimuli were too high in very small areas of the scaffold to lead to any cell differentiation.

## 4 Discussion

In this study, a transient one-way FSI model was developed to analyze the influence of mechanical stimulation on cell differentiation. After selecting a reliable simulation setup (e.g., ensuring the independency of results from mesh size and simulation time step size), the influence of the sinusoidal compression loads on cell differentiation was studied. At low compressive loads, the bone cell phenotype was predominantly predicted, which is also confirmed by previous research (Byrne et al., 2007; Milan et al., 2010; Castro and Lacroix, 2018; Horner et al., 2018). Increasing compression amplitudes resulted in differentiation of stem cells into cartilage and fibrous tissues, whereas the percentage of differentiated bone cells decreased.

The location of maximum strain was determined at the intersections of the strands, which was also found in previous studies (Hendrikson et al., 2017; Malve et al., 2018). It has been assumed that cell death may occur at OSS values higher than 0.225 (Olivares et al., 2009; Sandino and Lacroix, 2011). Our results report that the average values of OSS for all compression loads are below this value (Figure 4A). Considering the histogram for the highest compression amplitude of 10%, it can be seen that no region on the scaffold has an OSS higher than 0.225 (Figure 4D). Despite this fact, cell apoptosis occurs in a small area of the scaffold surface (Figure 7). This demonstrates that cell differentiation depends not only on the OSS, but also on the WSS caused by the fluid flow and both effects have to be considered simultaneously.

The acceptable WSS range for cell differentiation was assumed to be [0.01 60] mPa (Sandino and Lacroix, 2011), while a peak shear stress of 57 mPa was associated with cell apoptosis (Porter et al., 2005). Our analysis of ${WSS}_{avg}$ shows that the area-weighted average values of WSS for all load conditions are within the mentioned range (Figure 4B). Furthermore, the histogram for the 10% compression amplitude indicates that even in the worst case (highest values of WSS), only a very small area of the scaffold exhibits WSS values higher than the critical value of 57 mPa (Figure 4E).

Although *in vitro* and *in vivo* experiments have shown that dynamic compression promotes MSCs’ differentiation, the mechanism which explains the influence of compression stimulus on MSCs has not yet been wholly understood (Sun et al., 2022). Thus, selecting the appropriate simulation time point when the mechanical stimulation could determine the cell phenotype was one of the challenges in predicting tissue phenotype using the dynamic FSI model. Since the mechanical stimulation depends on both the WSS induced by fluid flow and the OSS due to the deformation of the scaffold, it was impossible to determine the maximum stimulus at each node during simulation. Each scaffold area experienced variable fluid shear stress stimuli and compression strain stimuli. The maximum values of these two stimuli types did not occur at the same time point. Other researchers also reported that certain scaffold areas are affected by maximum values of just one of these stimuli (Sandino et al., 2008; Malve et al., 2018). Therefore, averaged values of these parameters were investigated during one loading cycle. However, the maximum ${WSS}_{avg}$ and ${OSS}_{avg}$ did not appear simultaneously, as seen in Figure 5A. Figure 5 emphasizes the importance of the co-simulation method in simultaneously analyzing fluid-induced stimuli versus structural loading-induced stimuli. For example, Figure 5B displays that when ${OSS}_{avg}$ reached the highest value ( t=0.5 s in Figure 5A), the WSS distribution on the scaffold was negligible. In contrast, when ${WSS}_{avg}$ was at its maximum (t = 0.728 s in Figure 5A), OSS did not reach high values on the scaffold surface, as shown in Figure 5D. Considering these issues, the parameter $S_{avg}$ was defined based on ${WSS}_{avg}$ and ${OSS}_{avg}$ . The maximum value of $S_{avg}$ determined the simulation time at which cell differentiation was predicted (t = 0.656 s for the 10% compression in Figure 5A).

The literature describes that the magnitude of compression is a possible parameter to control MSC differentiation (Sun et al., 2022). Our study also demonstrated that the compression amplitude is a crucial factor for cell differentiation. For instance, when the compression amplitude was increased from 5% to 10%, the portion of bone cell phenotype on the scaffold surface decreased from 48.64% to 12.53%. Conversely, the fibrous cell phenotype showed an increase from 1.12% to 31.62% while no significant change in the proportion of cartilage cell phenotype was observed (Figure 7). Prediction of cell differentiation on a scaffold as a function of compression amplitude was previously investigated by Hendrikson et al. (2017). Their computed cell differentiation results, based on the combination of OSS and WSS for scaffolds with orthogonal strands (0/90 scaffold architecture), show qualitatively almost the same trend as the results in Figure 7. However, their computational analysis did not consider compression-induced fluid flow. In addition, their modeled scaffolds differed from the scaffolds in the current study, leading to quantitatively different results.

The combination of FE analysis and FVM within a transient FSI model allows us to obtain valuable data from both the fluid and solid domains. However, the accuracy of the model in predicting cell differentiation depends on the meshing strategy and the number of elements, since in FVM, the results are computed at element centers. In contrast, in our FE model, they are determined at element nodes and also at internal nodes for elements with Lagrangian multiplier (Ansys^®^ Academic Research Mechanical, 2020b). Therefore, transferring the FVM results from element centers to the element nodes is necessary to calculate stimuli, but this requires interpolating these results. A reduction of the element size is required to reduce the differences between nodal and central values of the CFD model. This leads to the use of a fine mesh for both the FE and CFD models, with the chosen conformal meshing approach being computationally expensive.

Although a one-way FSI model was applied in this study, fluid flow in other bioreactor configurations, such as perfusion bioreactors, may cause deformation of the scaffold, and this resulting displacement may also affect fluid motion. For such problems, two-way FSI simulations should be considered.

Since the material properties of the model did not change over time, the analysis of cell differentiation in a single loading cycle, as performed in this study, is sufficient. Due to the constant material properties, the prediction of the cell phenotypes does not change with the presented model when the simulation is performed for additional loading cycles. In other words, the model just considered the homeostatic state of the scaffold, and the intermediate situations that influence cell differentiation were neglected. In future analyses, the changes in material characteristics and scaffold morphology due to neo-tissue formation should be updated over the loading cycles during long-term stimulations. Hence, real *in vitro* changes in scaffold properties can be considered, and the model will make better predictions. Moreover, the hyperplastic material used cannot mimic all the properties of hydrogels. Therefore, the application of time-dependent parameters of hyper-viscoelastic material models can also improve the predictability of cell differentiation (Weizel et al., 2023). This will particularly be of interest as the viscoelastic properties, i.e., the stress relaxation behavior, has been shown to control cell behavior (Chaudhuri et al., 2015; Chaudhuri et al., 2016). Finally, even though our study predicts cell differentiation based on an experimentally validated mechano-regulation theory (Prendergast et al., 1997), a compression bioreactor could be utilized to validate the numerical results of our model.

## 5 Conclusion

In this study, a transient one-way FSI model for predicting cell differentiation on the surface of a mechanically stimulated porous hydrogel scaffold was presented. This model considers both stimulation due to mechanical deformation of the scaffold and due to compression-induced fluid flow during dynamic compressive stimulation. The presented model thus goes beyond previous FSI studies in the field of computational mechanobiology, which were mainly dealing with perfusion flows and steady-state-flow models. Stimulation due to structural strain and fluid shear stress stimuli were calculated simultaneously. It was shown that the amplitude of the compression load is a decisive factor for the control of cell differentiation. The results showed good agreement with previous studies and highlighted the applicability of the model in computational mechanobiology. This model can be used to not only analyze the influence of load amplitude but also of other stimulation parameters such as frequency, scaffold structure, and material properties on cell differentiation in early stages of cell cultivation. Consequently, the model can be used to optimize scaffold designs and stimulation protocols. Future studies should consider the change in material properties and tissue morphology that occur during long-term stimulation to improve the predictability of the model. For this purpose, the model parameters must be adjusted in simulations over several loading cycles. The FSI simulation is a powerful approach that can help to reduce *in vitro* and *in vivo* experiments and lower costs. In the field of mechanobiology, the presented approach can be applied in numerous other studies in the future to predict cell differentiation in similar tissue engineering problems based on mechanical stimulation.

## Data Availability

The original contributions presented in the study are included in the article/Supplementary Material, further inquiries can be directed to the corresponding author.
