# Supplementary material for: Simulating the mechanical stimulation of cells on a porous hydrogel scaffold using an FSI model to predict cell differentiation
Source: Front Bioeng Biotechnol. 2023 Sep 19;11:1249867. doi: 10.3389/fbioe.2023.1249867 (PMC10549991; doi:10.3389/fbioe.2023.1249867)
Supplement: Supplementary file 1 [file Table1.DOCX]

Supplementary Material

| Mesh Size | Number of FE Model Elements | Number of CFD Elements | Total Simulation time: CFD + FE Model + Coupling |
| --- | --- | --- | --- |
| **Coarse** | 311,445 | 612,786 | 14 hours |
| **Medium** | 780,094 | 1,216,293 | 2 days + 12 hours |
| **Fine** | 1,274,119 | 1,801,339 | 6 days + 17 hours |

**Table S1.** Mesh independency analysis with three different mesh configurations. All simulations were performed at 10% compression on the hardware described in Section 2.5 using parallel processing to reduce simulation time.

**Table S2.** Relative errors in cell phenotype prediction of coarse and medium mesh sizes compared to fine mesh size at three sample simulation times of 0.3s, 0.5s and 0.6s.

| Mesh Size | Relative Errors of Cell Prediction [%] | | | | | | | | |
| --- | --- | --- | --- | --- | --- | --- | --- | --- | --- |
|  | $\boldsymbol{t=0.3 s}$ | | | $\boldsymbol{t=0.5 s}$ | | | $\boldsymbol{t=0.6 s}$ | | |
|  | Bone | Cartilage | Fibrous | Bone | Cartilage | Fibrous | Bone | Cartilage | Fibrous |
| **Coarse** | -5.37 | 10.43 | -2.84 | -0.77 | -10.14 | 19.85 | 4.80 | 2.03 | -0.58 |
| **Medium** | -0.53 | 2.61 | 1.21 | -1.33 | -3.43 | 10.39 | 0.92 | 0.38 | 1.22 |
